# Correlations between angiogenic factors and capillaroscopic patterns in systemic sclerosis

**DOI:** 10.1186/ar4217

**Published:** 2013-04-19

**Authors:** Jérôme Avouac, Maeva Vallucci, Vanessa Smith, Patricia Senet, Barbara Ruiz, Alberto Sulli, Carmen Pizzorni, Camille Frances, Gilles Chiocchia, Maurizio Cutolo, Yannick Allanore

**Affiliations:** 1 Rheumatology A department, Paris Descartes University, Sorbonne Paris Cité, Cochin Hospital, rue du Faubourg Saint Jacques, 27, Paris, 75014, France; 2 INSERM U1016 and CNRS UMR8104, Cochin Institute, Paris Descartes University, rue du Faubourg Saint Jacques, 27, Paris, 75014, France; 3 Department of Rheumatology, Ghent University Hospital, De Pintelaan 185, Ghent, 9000 Belgium; 4 Department of Dermatology, Paris X University, Tenon Hospital, Rue de la Chine, 4, Paris, 75020, France; 5 Research Laboratory and Academic Unit of Clinical Rheumatology, Department of Internal Medicine, University of Genova, Viale Benedetto XV, 6, Genova, 16132, Italy

## Abstract

**Introduction:**

We sought to assess whether nailfold videocapillaroscopy (NVC) patterns are associated with levels of angiogenic factors in systemic sclerosis (SSc).

**Methods:**

Circulating endothelial progenitor cells (EPCs) and circulating endothelial cells (CECs) were measured in the peripheral blood of 60 consecutive SSc patients. Serum levels of eight endothelial markers were measured first in these 60 patients, and then in an independent replication cohort of 43 SSc patients in case of association with NVC patterns. NVC patterns were determined by four independent investigators blinded to vascular markers.

**Results:**

Patients with the late-NVC pattern exhibited lower EPC levels (*P *< 0.0001) and higher VEGF levels (*P *= 0.03). Higher VEGF levels were confirmed to be associated with the late-NVC pattern in the replication cohort (*P *= 0.01). By multivariate analysis focused on biomarkers, lower EPC (*P *= 0.03) and higher VEGF levels (*P *= 0.001) were independently associated with the late-NVC pattern. In an alternate multivariate model including these two factors and SSc-related disease characteristics, lower EPC counts (*P *= 0.005), higher VEGF levels (*P *= 0.01), a history of digital ulcers (*P *= 0.04), and a modified Rodnan skin score > 14 (*P *< 0.0001) were independently associated with the late-NVC pattern.

**Conclusion:**

Our data revealed decreased EPC counts and increased VEGF levels in patients with the late-NVC pattern. Further studies are now needed to determine the role of VEGF and EPCs in endothelial injury and repair in SSc.

## Introduction

Systemic sclerosis (SSc) is a severe connective tissue disease characterized by vascular, immune, and fibrotic changes in the skin and some internal organs [1]. Early and diffuse microvascular alterations are key features of SSc, with outcome depending on the extent and severity of vascular lesions. The earliest clinical symptoms of SSc relate to disturbances in the peripheral vascular system. Moreover, work with animal models showed that endothelial cell apoptosis could be a primary event in the pathogenesis of SSc [2-6]. Endothelial cell injury results in disorganization of endothelial layer favoring early impaired capillary architecture and loss of capillaries [7]. These features can be detected on nailfold videocapillaroscopy (NVC), which shows a variety of morphologic changes including enlarged capillaries, bushy capillary formations, microhemorrhages, and a variable loss of capillaries with or without avascular areas [8,9] from the very early stages. In accordance, NVC is used as a marker in early and even preclinical stages of SSc [10]. This decreased capillary density, insufficiently compensated by endothelial repair processes, which relate to angiogenesis and vasculogenesis, results in blood flow being insufficient and reduction of oxygen supply that leads to tissue hypoxia. These phenomena are often accompanied by abnormal levels of angiogenic/angiostatic factors and markers of endothelial cell injury, as well as disturbed vasculogenesis with abnormal numbers of circulating endothelial progenitor cells (EPCs) [11-17]. In addition, several vascular markers, including circulating EPCs, placenta growth factor (PlGF) and soluble vascular cell-adhesion molecule (sVCAM) have been recently shown to predict the occurrence of ischemic digital ulcers and cardiovascular events, complications that directly relate to microvascular complications [18].

Thus, we hypothesized that microvascular abnormalities with disturbed capillary architecture objectified on NVC could be related to an impairment of selective factors reflecting disturbances of angiogenesis, endothelium damage, and vasculogenesis. To test this hypothesis, we assessed whether NVC changes were associated with peripheral blood or serum levels of endothelial factors in SSc.

## Materials and methods

### Patient sets

Two different cohorts were used in this study. All patients gave informed consent for all procedures, which were carried out with local ethics committee approval (Comité de Consultation pour la Protection des Personnes se prêtant à la Recherche Biomédicale (CCPPRB) Paris-Cochin).

### Discovery cohort

The first patient set consisted on consecutive patients with SSc referred to the Rheumatology A Department over a 6-month period for systematic follow-up. Eligible patients were those who had been on a stable treatment regimen for at least 3 months, including vasodilators (calcium channel blockers, angiotensin-converting enzyme inhibitors, endothelin-receptor antagonists, PDE5 inhibitors) and immunosuppressive drugs (methotrexate, azathioprine, and prednisone dosage ≤ 10 mg/day). No patient was treated with cyclophosphamide or prostacyclins at the time of this study.

#### Clinical assessments

We systematically collected the following data: age, disease duration (date of the first non-Raynaud symptom), duration of Raynaud phenomenon, cutaneous subset according to the criteria reported by LeRoy *et al. *[19], skin involvement according to the modified Rodnan skin score (mRSS) [20], digital ulceration (past or current), and treatment received.

#### Routine laboratory assessments

Routine laboratory studies obtained on the morning of hospital admission included complete blood cell count, Westergren erythrocyte sedimentation rate (ESR), C-reactive protein (CRP) level, serum creatinine concentration, levels of proteinuria and von Willebrand factor antigen (ELISA, VIDAS von Willebrand; BioMérieux, Marcy l'Etoile, France), and tests for anticentromere (immunofluorescence on Hep2 cells) and antitopoisomerase-I antibodies (counter immunoelectrophoresis).

#### Pulmonary and cardiac assessments

Echocardiography was performed by a senior cardiologist according to the American Society of Echocardiography recommendations. In particular, left ventricular ejection fraction (LVEF) was determined by using the Simpson method, and systolic PAP (sPAP) was based on the tricuspid and/or pulmonary regurgitation, adding 10 mm Hg for auricular pressure. Pulmonary hypertension was suspected at baseline or during follow-up based on (a) an estimated echocardiographic systolic pulmonary arterial pressure (sPAP) > 40 mm Hg, Or (b) a DLCO < 50% predicted in absence of pulmonary fibrosis; or (c) unexplained dyspnea, as previously published [21,22] and had to be confirmed by RHC as a resting mean pulmonary artery pressure ≥ 25 mm Hg together with a pulmonary capillary wedge pressure of ≤ 15 mm Hg (precapillary PH).

Pulmonary involvement was assessed with chest radiograph, computed tomography, and measurement of forced vital capacity (FVC) and the diffusing capacity for carbon monoxide/alveolar volume (DLCO/AV) ratio.

### Replication cohort

This second patient set consisted on patients with SSc recruited during a patient meeting (business meeting of the Association des Sclérodermiques de France, Strasbourg, 27 April, 2012). Age, disease duration, and cutaneous subset according to the criteria reported by LeRoy *et al. *[19] were collected for all the patients, together with the main clinical complications and ongoing medications.

#### Nailfold videocapillaroscopy

NVC was performed for all patients of the two cohorts. Patients were asked not to smoke and take caffeinated drinks for at least 4 hours before NVC, and to remain at least 15 minutes at 22°C to 23°C before NVC.

#### Collection and blinding of the NVC images

The nailfolds of the second, third, fourth, and fifth fingers were examined bilaterally in each patient by two independent investigators blinded for the serum status of SSc patients (discovery cohort: JA, replication cohort: JA and PS), by using an optical probe videocapillaroscope equipped with a ×200 magnification contact lens and connected to image-analysis software (Videocaps, DS MediGroup, Milan, Italy). Four consecutive fields extending over 1 mm, in the middle of the nailfold, were studied per finger [23,24]. The images were made anonymous before being assessed by four independent investigators (VS, AS, CP, MC), blinded for the clinical and serum status of SSc patients.

#### Qualitative assessment of NVC images

The following NVC definitions were used for the qualitative assessment of the NVC patterns.

The early NVC scleroderma pattern: the combination of few enlarged/giant capillaries, few capillary microhemorrhages, a relatively well-preserved capillary distribution, and no evident loss of capillaries.

The active NVC scleroderma pattern: frequent giant capillaries, frequent capillary microhemorrhages, moderate loss of capillaries, mild disorganization of the capillary architecture, and absent or mild ramified capillaries.

The late-NVC scleroderma pattern: irregular enlargement of the capillaries, few or absent giant capillaries and microhemorrhages, severe loss of capillaries with large avascular areas, disorganization of the normal capillary array, and ramified/bushy capillaries (7). The Additional File 3 shows representative pictures of these three NVC patterns.

#### EPC and CEC cell sorting and flow-cytometry quantification

EPC and CEC quantification were performed only in the discovery cohort. Quantification of EPCs and CECs was precluded in the replication cohort because this procedure required fresh blood samples, and the patient meeting was organized in another city than the one where our laboratory is located. As used in previous reports, 20-ml samples of heparinized venous blood were taken at rest from the forearm, in the morning, at the same time as samples collected from hospitalized patients for routine analysis [15]. Samples were immediately transported to the laboratory for testing. We used a method previously described and validated to enrich mononuclear cells and quantify EPCs and CECs [15,18,25]. Negative lineage (Lin-) mononuclear cells were obtained by enrichment from 20-ml peripheral blood mononuclear cells by using 1,000 ml of a human progenitor cell enrichment cocktail (RosetteSep; StemCell Technologies, Vancouver, BC, Canada). The enriched mononuclear cells were collected by Ficoll density gradient centrifugation (Pancoll; Dutcher, Brumath, France), and then washed and preincubated for 15 minutes with 100 ml of an FcR blocking reagent (Miltenyi Biotec, Paris, France) to inhibit nonspecific binding or specific binding via Fc receptors. These cells were then subjected to triple labeling for EPC or CEC detection, according to recent recommendations and previous publications [15]. For EPC detection, 10 ml of enriched mononuclear cells was labeled with 5 ml of each anti-CD133-phycoerythrin (PE, Miltenyi Biotec) and anti-CD34-fluorescein isothiocyanate (FITC; BD Bioscience, Le Pont de Claix, France) antibodies, and 20 ml of anti-VEGFR-2 (KDR)-allophycocyanin (APC; R&D Systems, Minneapolis, MN, USA) antibodies. For CEC detection, 10 ml of enriched mononuclear cells was labeled with 5 ml each of anti-CD133-PE and anti-CD105-FITC (AbD Serotec, Colmar, France) and 20 ml of anti-VEGFR-2-APC. Five milliliters of 7AAD (BD Bioscience) were used for real-time viability staining to identify dead cells 20 minutes before flow cytometry. Control cells were also prepared by incubation with fluorescence-labeled isotype-matched monoclonal antibodies (Beckman Coulter, Villepinte, France). The labeled cells were analyzed by using a fluorescence-activated cell sorter (FACS; four-color flow cytometry with a FACS Calibur flow cytometer (BD Bioscience). A large number of events (500,000) was considered for every sample. The same detector sensitivity, compensation setting, and scatter-gate set were used to analyze all samples. Viable cells were identified by gating on forward/side scatters and as the 7AAD-population. The expression of CD133 and VEGFR-2 by gated viable CD34^+ ^cells was assessed. The EPC and CEC populations were identified as Lin^-^/7AAD^-^/CD133^+^/CD34^+^/VEGFR-2^+^, and Lin^-^/7AAD^-^/CD133^-^/CD105^+^/VEGFR-2^+ ^cells, respectively. Data were analyzed with FlowJo software (Version 7.6.5). Results were expressed as the number of EPCs per million Lin- mononuclear cells.

### Serum levels of endothelial markers

Serum was isolated from the peripheral blood of all patients included in the discovery and replication cohorts. In the replication cohort, sera was also taken at rest from the forearm, in the morning, and stored locally before shipment to our laboratory, in the days after the patient meeting. Serum levels of eight endothelial factors that have been shown to be implicated in SSc (VEGF, placenta growth factor (PlGF), sVCAM, angiopoietin-2, endoglin, endostatin, endothelin-1 and Tie-2) were measured by using the quantitative sandwich enzyme-linked immunosorbent assay (ELISA) technique (Quantikine kits; R&D systems) [11-14,17,18,26-28]. No patient in our cohort had regular alcohol consumption, which has been reported to have an important influence on serum levels of VEGF. Concentrations were calculated by using a standard curve generated with specific standards provided by the manufacturer. The analytic range, and intraassay and interassay coefficients of variation are detailed for each marker in Additional File 1. Serum levels of these eight endothelial markers were first assessed in the discovery cohort. Only serum levels of endothelial markers that were found associated with NVC patterns in the discovery cohort were measured in the replication cohort.

### Statistical analysis

All data are presented as median (range) for continuous variables and numbers and percentages for categoric variables, unless stated otherwise. The χ^2 ^test was used to compare categoric variables. In that case, patients with early and active NVC patterns were merged and further compared with patients with the late-NVC pattern. Comparisons between the three NVC patterns were by a nonparametric Kruskal-Wallis test. A multiple linear regression analysis was also performed for all variables identified with *P *≤ 0.1 univariately. *P *values < 0.05 were considered significant.

## Results

### Study population

The discovery cohort consisted of 60 consecutive SSc patients (46 women, 77%), with a median (range) age of 53.5 years (28 to 81 years) and median (range) disease duration of 9 years (1 to 50 years). Thirty-six (60%) patients had the diffuse cutaneous subset, and 24, the limited. Their baseline characteristics are shown in Table 1.

**Table 1:** Characteristics of the 60 patients with systemic sclerosis included in the discovery cohort

Age (years), median (range)	53.5 (28-81)
Disease duration (years), median (range)	9 (1-50)
Limited/Diffuse cutaneous subset, *n *(%)	36 (60)/24 (40)
Modified Rodnan skin score > 14, *n *(%)	19 (32)
History of digital ulcers, *n *(%)	29 (47)
Pulmonary fibrosis on CT scan, *n *(%)	28 (47)
Pulmonary arterial hypertension on RHC, *n *(%)	4 (7)
Positive antinuclear antibodies (> 1/160), *n *(%)	53 (88)
Positive antitopoisomerase-1 antibodies, *n *(%)	28 (47)
Positive anticentromere antibodies, *n *(%)	12 (20)
FVC < 75% predicted, *n *(%)	13 (22)
DLCO/AV < 75% predicted, *n *(%)	23 (38)
Treatment with calcium channel blockers, *n *(%)	60 (100)
Treatment with angiotensin-converting enzyme inhibitors, *n *(%)	19 (32)
Treatment with endothelin-receptor antagonists and/or PDE5 inhibitors, *n *(%)	12 (20)

DLCO/AV, diffusing capacity for carbon monoxide/alveolar volume; FVC, forced vital capacity; PDE5, phosphodiesterase type 5; RHC, right heart catheterization.

The replication cohort consisted on 43 patients with SSc (33 women, 77%), with a median (range) age of 57 years (34 to 80 years) and median (range) disease duration of 7 years (1 to 29 years). Fifteen (35%) patients had the diffuse cutaneous subset, and 28, the limited.

### Qualitative capillaroscopic assessment

Fourteen (23%) patients had an early-, 22 (37%), an active-, and 24 (40%), a late-NVC SSc pattern in the discovery cohort. In the replication cohort, eight (19%) patients had an early-, 20 (46%) an active-, and 15 (35%) a late-NVC pattern.

### Levels of endothelial factors

To assess the validity of flow cytometry and ELISA measurements, we compared the levels of endothelial markers observed in SSc patients with the values obtained in a population of 20 healthy age- and sex-matched controls [15,29]. EPC levels were significantly higher than those of controls, in agreement with our previous results [15], and CEC levels were significantly decreased in SSc patients (see Additional file 2). Serum levels of VEGF, PlGF, endostatin, endothelin-1, and angiopoietin-2 were significantly higher in SSc patients; and no difference was observed with controls for sVCAM, endoglin, and Tie-2 serum levels (Additional file 2).

### Levels of endothelial factors and NVC patterns

**Figure 1:**
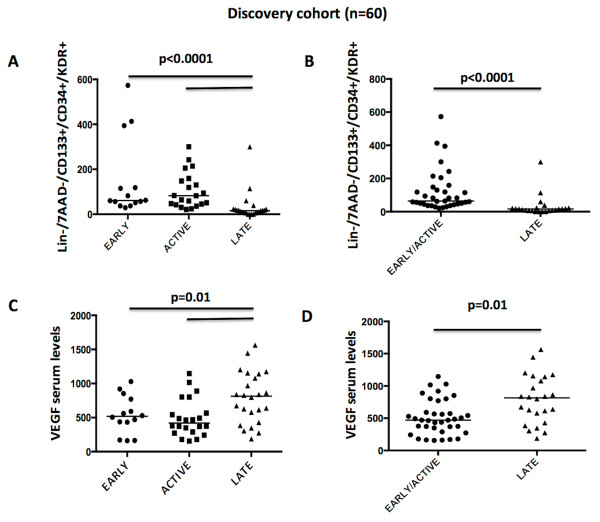
**Levels of circulating endothelial progenitor cells (Lin-7AAD-CD133^+^CD34^+^VEGFR2^+ ^cells) (A, B) and VEGF serum levels (C, D) in the discovery cohort according to the nailfold videocapillaroscopy pattern**.

In univariate analysis focused on vascular factors, patients from the discovery cohort and with the late-NVC pattern exhibited significantly lower EPC levels than did patients with early and active patterns (median EPC levels, 31/10^6 ^Lin-mononuclear cells in the late-NVC pattern versus 61 and 82/10^6 ^Lin-mononuclear cells in the early- and active-NVC patterns, respectively; *P *< 0.0001) (Table 2 and Figure 1A, B).

**Table 2:** Nailfold videocapillaroscopy patterns and endothelial marker levels in peripheral blood or serum of patients with systemic sclerosis included in the discovery cohort

	Capillaroscopy pattern			*P *value
		
	Early Median (range)	Active Median (range)	Late Median (range)	
EPCs (10^6 ^Lin-mononuclear cells)	61 (29-573)	82 (21-300)	31 (5-300)	< 0.0001
CECs (10^6 ^Lin-mononuclear cells)	81 (10-342)	36 (5-155)	77 (6-472)	0.4
VEGF (pg/ml)	518 (161-1,028)	493 (156-1,146)	814 (189-1,564)	0.01
PlGF (pg/ml)	9.6 (4.6-24.2)	9.9 (0.7-19.6)	10.5 (2.3-26.5)	0.8
sVCAM-1 (ng/ml)	766 (482-1,828)	694 (354-1,405)	767 (459-1,314)	0.4
Tie-2 (ng/ml)	24.4 (12.9-29.4)	21.1 (14.1-27.1)	22.2 (16-39.6)	0.3
Endostatin (ng/ml)	185 (88-477)	139 (54-178)	150 (22-662)	0.3
Endoglin (CD105) (ng/ml)	3.7 (1.9-5.0)	3.9 (2.8-5.6)	3.5 (2.6-5.1)	0.5
Endothelin-1 (pg/ml)	1.5 (1.0-3.6)	2.5 (0.9-7.2)	1.7 (0.4-3.1)	0.02
Angiopoietin-2 (pg/ml)	2,166 (1241-3,312)	2,016 (1,249-4,027)	2202 (1,512-5,148)	0.5
von Willebrand factor antigen (%)	180 (95-266)	169 (69-304)	170 (83-302)	0.9

**Figure 2:**
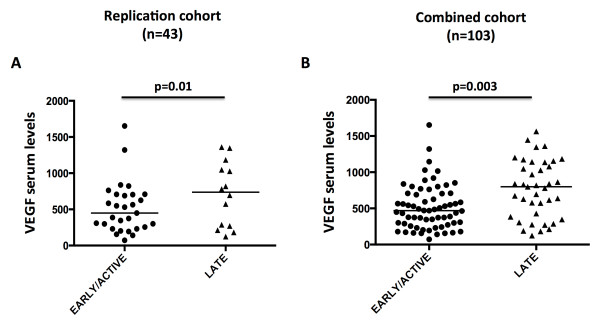
**Serum VEGF levels in the replication cohort (A), and combined cohort (B), according to the nailfold videocapillaroscopy pattern**.

In the discovery cohort, higher VEGF serum levels were observed in the late-NVC pattern (median VEGF serum levels, 814 pg/ml), as compared with the early- (518 pg/ml) and active- (493 pg/ml) NVC patterns (*P *= 0.01) (Table 2 and Figure 1C, D). This result was confirmed in the replication cohort (median VEGF levels in the late-NVC pattern: 708 pg/ml versus 445 pg/ml in patients with the early- or active-NVC pattern; *P *= 0.01) (Figure 2A), and in the combined population of 103 patients (median VEGF levels in the late-NVC pattern: 770 pg/ml versus 483 pg/ml in patients with the early- or active-NVC pattern; *P *= 0.003) (Figure 2B).

Endothelin-1 serum levels were significantly higher in the active pattern (2.5 pg/ml) compared with the early and late patterns (1.5 and 1.7 pg/ml; *P *= 0.02) in the discovery cohort (Table 2). This result was not confirmed in the replication cohort (median endothelin-1 levels in the active NVC pattern: 2.9 pg/ml versus 2.1 pg/ml in patients with the early-NVC pattern and 3.2 pg/ml in patients with the late-NVC pattern; *P *= 0.2), or in the combined cohort.

No association was observed between the levels of other serum markers and NVC patterns in the discovery cohort (Table 2).

In multivariable analysis performed with multiple linear regression, including EPC counts, VEGF serum levels, and endothelin serum levels, only EPC counts (*r *= -0.41; *P *= 0.003) and VEGF serum levels (*r *= 0.36; *P *= 0.001) were independently associated with the late-NVC pattern.

### SSc disease characteristics and NVC patterns in the discovery cohort

The χ^2 ^analysis performed revealed several associations between the late-NVC pattern and SSc-related disease characteristics (Table 3). Patients with the late-NVC pattern were more likely to have an mRSS > 14 (13 of 24, 54%, versus six of 36, 17%; *P *= 0.005) and a history of digital ulcers (16 of 24, 67%, versus 13 of 36, 36%; *P *= 0.03) than patients with the early- or the active-NVC pattern. In addition, the likelihood of positive antitopoisomerase-I antibodies (16 of 24, 67%, versus 12 of 36, 33%; *P *= 0.02) and decreased DLCO/AV < 75% (14 of 24, 58%, versus nine of 36, 25%; *P *= 0.02) was significantly higher in patients with the late-NVC pattern.

**Table 3:** Association between nailfold videocapillaroscopy patterns and disease characteristics of patients with systemic sclerosis included in the discovery cohort

	NVC pattern		*P *value	Multiple linear regression (*r*)	*P *value
				
	Early/active (*n *= 36)	Late (*n *= 24)			
Age, median (range)	58	53	0.2		
Females, *n *(%)	27 (75)	19	0.9		
Disease duration, median (range)	9.91	7.20	0.2		
Diffuse cutaneous subset, *n *(%)	13 (36)	11 (46)	0.9		
Modified Rodnan skin score > 14, *n *(%)	6 (17)	13 (54)	0.005*	0.42	0.0008
History of digital ulcers, *n *(%)	13 (36)	16 (67)	0.03*	0.25	0.03
Pulmonary fibrosis, *n *(%)	15 (42)	13 (54)	0.5		
Pulmonary arterial hypertension on RHC, *n *(%)	2 (6)	2 (8)	0.9		
Positive antitopoisomerase antibodies, *n *(%)	12 (33)	16 (67)	0.02*		
Positive anticentromere antibodies, *n *(%)	9 (25)	3 (13)	0.4		
FVC < 75, *n *(%)	5 (14)	8 (33)	0.1*	0.29	0.01
DLCO/VA < 75%, *n *(%)	9 (25)	14 (58)	0.02*		
Treatment with angiotensin-converting enzyme inhibitors, *n *(%)	8 (22)	11 (46)	0.09*		
Treatment with endothelin-receptor antagonists and/or PDE5 inhibitors, *n *(%)	4 (11)	8 (33)	0.08*		

DLCO/AV, diffusing capacity for carbon monoxide/alveolar volume; FVC, forced vital capacity; NVC, nailfold videocapillaroscopy; PDE5, phosphodiesterase type 5; RHC, right heart catheterization.

* Variables included in multiple regression analysis

In multiple linear regression analysis performed for all variables identified with *P *≤ 0.10 univariately, an mRSS > 14 (*r *= 0.42; *P *= 0.0008), history of digital ulcers (*r *= 0.25; *P *= 0.03), and an FVC < 75% (*r *= 0.29; *P *= 0.01) were independently associated with the late-NVC pattern (Table 3).

### Angiogenic factors, SSc disease characteristics, and NVC patterns

We next tested an alternate multiple linear regression model including both angiogenic markers independently associated with the late-NVC pattern (circulating EPCs and VEGF serum levels) and SSc-related disease characteristics associated univariately with *P *≤ 0.10 with the late-NVC pattern.

In this model, lower EPC counts (*r *= -0.45; *P *= 0.005) and higher VEGF levels (*r *= 0.37; *P *= 0.01) remained independently associated with the late-NVC pattern, together with a modified Rodnan skin (mRSS) score > 14 (*r *= 0.50; *P *< 0.0001) and history of digital ulcerations (*r *= 0.25; *P *= 0.04) (Table 4).

**Table 4:** Alternate multiple linear regression model performed in the discovery cohort and including both angiogenic factors independently associated with the late-NVC pattern and SSc-related disease characteristics associated univariately with *P *≤ 0.10 with the late-NVC pattern

	NVC pattern		*P *value	Multiple linear regression (*r*)	*P value*
				
	Early/Active (*n *= 36)	Late (*n *= 24)			
Modified Rodnan skin score > 14, *n *(%)	6 (17)	13 (54)	0.005*	0.50	< 0.0001
History of digital ulcers, *n *(%)	13 (36)	16 (67)	0.03*	0.25	0.04
Positive antitopoisomerase antibodies, *n *(%)	12 (33)	16 (67)	0.02*		
FVC < 75, *n *(%)	5 (14)	8 (33)	0.1*		
DLCO/VA < 75%, *n *(%)	9 (25)	14 (58)	0.02*		
Treatment with angiotensin-converting enzyme inhibitors, *n *(%)	8 (22)	11 (46)	0.09*		
Treatment with endothelin-receptor antagonists and/or PDE5 inhibitors, *n *(%)	4 (11)	8 (33)	0.08*		
EPCs (10^6 ^Lin-mononuclear cells), median (range)	64 (21-573)	31 (5-300)	< 0.0001*	-0.45	0.005
VEGF (pg/ml), median (range)	470 (156-1,146)	814 (189-1,564)	0.01*	0.37	0.01

DLCO/AV, diffusing capacity for carbon monoxide/alveolar volume; FVC, forced vital capacity; NVC, nailfold videocapillaroscopy. * Variables included in multiple regression analysis

## Discussion

This is the first study to show decreased EPC counts and increased VEGF serum levels in patients with the late-NVC pattern.

Reduced EPC numbers in the late-NVC pattern suggest that deficient vasculogenesis may contribute to the severe loss of capillaries observed in this pattern. Indeed, decreased EPC numbers can lead to insufficient endothelial repair and depressed new blood vessels formation that could be related to the extensive areas of desertification. Decreased EPC levels in this stage may be consistent with decreased mobilization from the bone marrow, as suggested in a previous study [30]. In the latter study, the stromal compartment of SSc bone marrow was defective in functional EPCs, particularly in patients with late-phase disease. Decreased EPC levels in the peripheral blood of patients with the late-NVC pattern could also be related to an increased EPC homing. Several studies previously reported that EPCs might be recruited at injured sites during active vascular and severe disease [15,31,32]. These data support overall insufficient vasculogenesis to counterbalance vascular damage and might suggest the mobilization of EPCs from the bone marrow (for example, with administration of G-CSF, statins, or erythropoietin) as a potential target for future therapies in SSc, especially in patients with the late-NVC pattern [33-35].

The severe capillary loss observed in the late-NVC pattern might also be related to decreased angiogenesis. This insufficient angiogenesis might be related to decreased levels of proangiogenic factors. However, we detected in two independent and homogeneous cohorts increased VEGF serum levels in SSc patients with the late-NVC pattern, compared with patients with the early- or active-NVC pattern. Thus, VEGF upregulation may appear as an insufficient compensatory mechanism to stimulate angiogenesis. In addition, the observation of lower EPC counts in the late-NVC pattern also supports that vasculogenesis is not sufficiently compensated by VEGF upregulation. This result is in accordance with those of other research groups that found significantly higher VEGF levels in the late stages of the disease, as compared with the levels in patients with recent onset [12,31]. Moreover, an inverse correlation between serum VFGF levels and capillary density has been reported [32,36]. The same findings have been observed in systemic lupus erythematosus: significantly higher VEGF serum levels have been detected in patients with decreased capillary density and neoangiogenesis (> 75% morphologically changed loops, as tortuous, enlarged, and/or disarranged capillaries) [37]. In addition, a prolonged overexpression of VEGF may have deleterious effects on the vascular network, because it may result in a chaotic vascular morphology with reduced blood flow in the newly formed vessels. A chronic and uncontrolled overexpression of VEGF does occur in SSc and might significantly be implicated in the altered vessel morphology observed in the late-NVC pattern [38].

We did not find any differential concentration of other proangiogenic markers (PlGF, angiopoietin-2, and Tie-2) between the different NVC patterns. One recent previous study identified a trend for higher angiopoietin-2 levels in patients with the late-NVC pattern with respect to those with an early/active pattern [17]. This discrepancy may be explained by the characteristics of the study populations (lower proportion of patients with the diffuse cutaneous subset and higher disease duration in our study) and by the measurement of angiopoietin-2 in the serum in our study, versus plasma in the other study. The insufficient angiogenesis observed in the late-NVC pattern might also be related to increased levels of angiostatic factors, which have been reported to be predominant in the late stages of SSc. However, we did not observe increased levels of endostatin and endoglin, two major inhibitors of angiogenesis. Further evaluation of other angiostatic markers should be assessed to confirm these findings.

We also determined whether SSc-disease characteristics might be associated with NVC patterns. No association was noted between the late-NVC pattern and disease duration, which supports that patients with early disease may experience severe vascular loss not sufficiently compensated by new-vessel formation. We found that the late-NVC pattern was associated in univariate analysis with more-severe vascular manifestations (history of digital ulcers) or with the presence of markers of a more-diffuse SSc subset (mRSS > 14 and antitopoisomerase-I antibodies). Multivariate analysis confirmed the independent association between the late-NVC pattern and the following characteristics: history of digital ulcers, mRSS > 14, and an FVC < 75% of predicted; which support a more-severe and fibrotic propensity of the late-NVC pattern. These results are consistent with previously published data, which showed that patients with the late-NVC pattern have an increased risk to experience digital ulcers, more-severe skin thickening, and decreased FVC and DLCO, compared with patients with early and active patterns [39]. In addition, an mRSS > 14 remained independently associated with the late-NVC pattern after the inclusion of the endothelial markers in the statistical model. These results support the more severe and fibrotic propensity of the late-NVC pattern.

Our study has several limitations that deserve consideration. Our study is limited by its observational design, and any pathogenic link emerging from this type of study should be taken very cautiously. Our sample size was too limited to assess adequately disease phenotype associations in specific subsets of patients, especially those with confirmed pulmonary arterial hypertension, or those with active digital ulcers. In addition, our NVC assessment was only qualitative, based on pattern recognition. Next to associations between endothelial factors and qualitative NVC assessment, efforts should be further made to find associations with quantitative assessment, based on counting of hallmark parameters of the SSc pattern. Now quantitative assessment has to find a definite place in research settings and also in daily practice. Thus, further evaluation of endothelial markers with some recently proposed quantitative index/scores might be performed to confirm our findings obtained with pattern recognition [40-42].

## Conclusions

Our data revealed decreased EPC counts and VEGF upregulation in patients with the late-NVC pattern. Further studies are now needed to determine the respective role of EPCs and VEGF in endothelial injury and endothelial repair in SSc.

## Abbreviations

CEC: circulating endothelial cell; CRP: C-reactive protein; DLCO/AV: diffusing capacity for carbon monoxide/alveolar volume; EPC: endothelial progenitor cell; ESR: erythrocyte sedimentation rate; mRSS: modified Rodnan skin score; NVC: nailfold videocapillaroscopy; PlGF: placenta growth factor; sPAP: systolic pulmonary artery pressure; SSc: systemic sclerosis; sVCAM: soluble vascular cell adhesion molecule; VEGF: vascular endothelial growth factor.

## Competing interests

Prof. Allanore and Dr. Avouac have received research grants and honoraria from Actelion and Pfizer (less than $10,000 USD each). The remaining authors have no competing interest.

## Authors' contributions

JA participated in the design of the study, performed nailfold videocapillaroscopy, participated to the analysis and interpretation of the data, performed the statistical analysis, and wrote the manuscript. MV performed the EPC/CEC quantification by flow cytometry and ELISA measurement, and collected the clinical data. VS participated in the analysis and interpretation of the data. PS performed nailfold videocapillaroscopy. BR performed the EPC/CEC quantification with flow cytometry and ELISA measurement. AS, CP, CF, GC, and MC participated in the analysis and interpretation of the data. YA conceived the study, participated in its design, participated in the analysis and interpretation of the data, and performed the statistical analysis. All authors drafted, read, and approved the final manuscript.

## Supplementary Material

Additional file 1 **Table S1**. Analytic range, and intraassay and interassay coefficients of variation of the quantitative sandwich enzyme-linked immunosorbent assay for each endothelial marker. Click here for file

Additional file 2 **Table S2**. Levels of the different endothelial markers in patients with systemic sclerosis in the discovery cohort: comparison with a population of 20 healthy controls issued from previous publications. Click here for file

Additional file 3 **Figure S1**. Representative pictures of the three nailfold videocapillaroscopy (NVC) patterns, as compared with a normal examination. Click here for file
