# Supplementary material for: Correlations between angiogenic factors and capillaroscopic patterns in systemic sclerosis
Source: Arthritis Res Ther. 2013 Apr 19;15(2):R55. doi: 10.1186/ar4217 (PMC4060197; doi:10.1186/ar4217)
Supplement: Additional file 1 — Table S1. Analytic range, and intraassay and interassay coefficients of variation of the quantitative sandwich enzyme-linked immunosorbent assay for each endothelial marker. [file ar4217-S1.DOCX]

**Additional file 1, Table S1: Analytical range, intra-assay and inter-assay coefficients of variation of the quantitative sandwich enzyme-linked immunosorbent assay for each endothelial marker**

| Endothelial marker | Analytical range | Intra-assay coefficient of variation (%) | Inter-assay coefficient of variation (%) |
| --- | --- | --- | --- |
| VEGF (pg/ml) | 62-707 | 5.1-6.7 | 6.2-8.8 |
| PlGF (pg/ml) | Undetectable-26 | 5.6-7.0 | 10.9-11.8 |
| sVCAM-1 (ng/ml) | 349-991 | 2.3-3.6 | 5.5-7.8 |
| Tie-2 (ng/ml) | 18.6-75.3 | 4.4-5.0 | 5.2-8.3 |
| Endostatin (ng/ml) | 58-232 | 3.6-6.9 | 5.7-7.9 |
| Endoglin (CD105) (ng/ml) | 2.54-7.06 | 2.8-3.2 | 6.3-6.7 |
| Endothelin-1 (pg/ml) | 0.472-2.00 | 1.9-4 | 5.3-7.6 |
| Angiopoietin-2 (pg/ml) | 1065-8907 | 4.2-6.9 | 7.4-10.4 |
