# Supplementary material for: Correlations between angiogenic factors and capillaroscopic patterns in systemic sclerosis
Source: Arthritis Res Ther. 2013 Apr 19;15(2):R55. doi: 10.1186/ar4217 (PMC4060197; doi:10.1186/ar4217)
Supplement: Additional file 2 — Table S2. Levels of the different endothelial markers in patients with systemic sclerosis in the discovery cohort: comparison with a population of 20 healthy controls issued from previous publications. [file ar4217-S2.DOCX]

**Additional file 2, Table S2: Levels of the different endothelial markers in patients with systemic sclerosis systemic in the discovery cohort: Comparison with a population of 20 healthy controls issued from previous publications**

| Endothelial marker | Patients  (n=60) | Controls  (n=20) | p-value |
| --- | --- | --- | --- |
| Endothelial progenitor cells (10^6^ Lin-mononuclear cells ) | 51 (5-573) | 27 (5-109) | 0.03 |
| Circulating endothelial cells (10^6^ Lin-mononuclear cells ) | 62 (5-472) | 154 (30-425) | 0.004 |
| VEGF (pg/ml) | 552 (156-1564) | 391 (74-653) | 0.007 |
| PlGF (pg/ml) | 10.0 (0,7-26,5) | 5.3 (0-11.5) | <0.0001 |
| sVCAM-1 (ng/ml) | 754 (354-1828) | 747 (310-1109) | 0.9 |
| Tie-2 (ng/ml) | 22.7 (12.9-39.6) | 25.1 (16.1-41.0) | 0.05 |
| Endostatin (ng/ml) | 146 (22-662) | 109 (68-464) | 0.009 |
| Endoglin (CD105) (ng/ml) | 3.7 (1.9-5.6) | 3.8 (2.9-4.7) | 0.8 |
| Endothelin-1 (pg/ml) | 1.7 (0.4-7.2) | 1.4 (0.5-2.4) | 0.01 |
| Angiopoietin-2 (pg/ml) | 2095 (1241-5148) | 1770 (1013-2827) | 0.01 |

All values are expressed as median (range)
