# Supplementary figures and images for: Correlations between angiogenic factors and capillaroscopic patterns in systemic sclerosis
Source: Arthritis Res Ther. 2013 Apr 19;15(2):R55. doi: 10.1186/ar4217 (PMC4060197; doi:10.1186/ar4217)

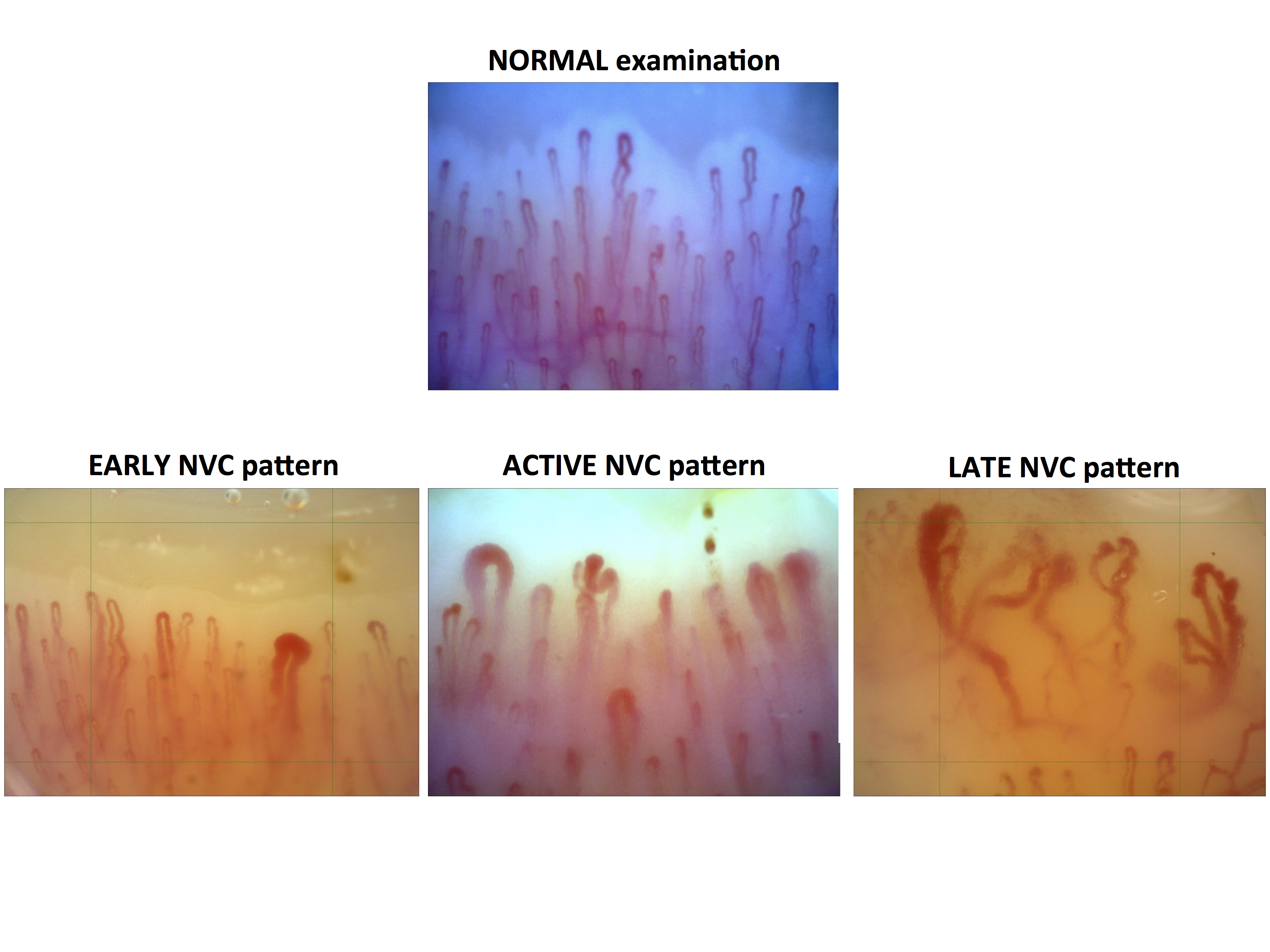

Supplement: Additional file 3 — Figure S1. Representative pictures of the three nailfold videocapillaroscopy (NVC) patterns, as compared with a normal examination. [file ar4217-S3.TIFF]
